# Nasal Delivery of Asiatic Acid Ameliorates Scopolamine-Induced Memory Dysfunction in Mice

**DOI:** 10.1155/2024/9941034

**Published:** 2024-09-09

**Authors:** Su Lwin Lwin Myint, Ratchanee Rodsiri, Hattaya Benya-Aphikul, Tissana Rojanaratha, Garnpimol Ritthidej, Ridho Islamie

**Affiliations:** ^1^ Department of Pharmacology and Physiology Faculty of Pharmaceutical Sciences Chulalongkorn University, Bangkok 10330, Thailand; ^2^ Preclinical Toxicity and Efficacy Assessment of Medicines and Chemicals Research Unit Chulalongkorn University, Bangkok 10330, Thailand; ^3^ Department of Pharmaceutics and Industrial Pharmacy Faculty of Pharmaceutical Sciences Chulalongkorn University, Bangkok 10330, Thailand; ^4^ Queen Saovabha Memorial Institute The Thai Red Cross Society, Bangkok 10330, Thailand; ^5^ Department of Clinical and Community Pharmacy Faculty of Pharmacy University of Surabaya, Surabaya 60293, Indonesia

## Abstract

Asiatic acid (AA) has previously shown its neuroprotective effects, but low oral bioavailability limits its penetration into the brain. This study aimed to investigate the effect of intranasal AA administration in mice with memory dysfunction induced by scopolamine. Mice received either intranasal AA (INAA), oral AA (POAA3 or POAA30), or donepezil, followed by scopolamine for 10 days. Morris water maze (MWM) was performed on days 0–5, 30 min after treatment. Locomotor activity was conducted on day 6 followed by brain collection. In MWM, INAA treatment had significantly reduced escape latency on days 2–4, while POAA3 decreased escape latency on day 3 and POAA30 and donepezil decreased escape latency on day 4. INAA inhibited acetylcholinesterase activity, increased catalase protein expression, and decreased malondialdehyde levels in the brain tissue. Therefore, intranasal administration of AA produced a rapid onset in the protection of learning and memory deficits induced by scopolamine through acetylcholinesterase inhibition and antioxidant effect.

## 1. Introduction

Alzheimer's disease (AD) is one of the most common neurodegenerative diseases in elderly patients that is often associated with learning deficits and memory loss [1]. Aggregated amyloid-beta (A*β*) plaque is one of the main pathological features of AD [2, 3]. Some evidence suggests that A*β* induces the formation of neurofibrillary tangles (NFTs) by promoting tau hyperphosphorylation of the nerve microtubule [2, 4]. NFTs, together with A*β*, could lead to neuronal loss in the hippocampus and induce neuroinflammation, oxidative stress, and cortical atrophy, and a decrease in cholinergic transmission [2, 5]. Current AD therapies such as donepezil and memantine are limited to symptomatic treatment, as they do not exhibit the protective effect to prevent the progression of AD [6]. Therefore, finding alternative treatments that can prevent the progression of AD has become increasingly important.

Asiatic acid (AA) is a bioactive substance that can be extracted from *Centella asiatica*. AA has been found to exhibit protective activity against neurotoxicity in the neuronal cell line exposed to glutamate and methamphetamine [7, 8]. In addition, AA prevented memory dysfunction induced by valproate, 5-fluorouracil, quinolinic acid, and AlCl_3_ [9–11]. The neuroprotective effects of AA involved its antioxidant activity [10, 12], anti-inflammation [12], and protection of adult neurogenesis [10, 11]. Moreover, *in vitro* and *in silico* studies revealed that AA can inhibit AChE [13]. Inclusively, AA is a promising compound in the treatment of AD.

AA has limitations in its pharmacokinetic profile when given orally. The bioavailability of AA is as low as 16.25% in rodents by oral administration, which could be attributed to the intensive metabolism by cytochrome C450 [14]. AA was encapsulated in the solid lipid nanoparticle (SLN) for intranasal delivery to increase AA penetration into the brain. Intranasal administration is seen to increase drug availability in the brain by avoiding first-pass metabolism and providing rapid onset of action, thus reducing systemic adverse effects [15]. Several CNS drugs such as carbamazepine, venlafaxine, olanzapine, and donepezil have been modified for the nasal delivery system [15–18]. Furthermore, due to the small particle size and lipophilic property, the solid lipid nanoparticles can be delivered to the brain via the olfactory and trigeminal nerves as well as through the blood-brain barrier [19].

This study aimed to determine the effect of intranasal AA administration on scopolamine-induced memory impairment in mice. In addition, the protective mechanisms of AA involving AChE activity, antioxidant enzyme, and lipid peroxidation were also examined.

## 2. Materials and Methods

### 2.1. Chemicals and Reagents

Asiatic acid (Sigma-Aldrich, St. Louis, MO, USA) was prepared in the SLN formulation for intranasal delivery. AA concentration in the SLN formulation was 2.26 mg/mL. The particle size, polydispersity index, and zeta potential of AA in SLN were 189.27 ± 4.22 nm, 0.321 ± 0.047, and −18.33 ± 0.45 mV, respectively [20]. AA at a concentration of 3 and 30 mg/mL was prepared in 0.5% carboxymethylcellulose (CMC) for oral administration. Scopolamine, donepezil, CMC, pyridine, 1,1,3,3-tetraethoxypropane (TEP), sodium dodecyl sulfate (SDS), thiobarbituric acid (TBA), and butanol were obtained from Sigma-Aldrich (St. Louis, MO, USA).

### 2.2. Animals

Male ICR mice (6–8 weeks old, 20–25 g) were obtained from the National Laboratory Animal Center (Mahidol University, Nakhon Pathom, Thailand) and acclimatized for 1 week before the experiments. The animals were kept under a 12-hour light-dark cycle with 22 ± 2°C of room temperature and 40–60% humidity. The mice were provided unrestricted access to both food and water. The experimental protocols were approved by the Institutional Animal Care and Use Committee, Faculty of Pharmaceutical Sciences, Chulalongkorn University, Thailand (approval no. 1933012). The ARRIVE guidelines were applied in all animal protocols.

### 2.3. Treatments

Mice were divided into six groups: control (CON), scopolamine (SCO), scopolamine plus donepezil (SCO + DON), scopolamine plus AA intranasal administration (SCO + INAA), and two groups for scopolamine plus AA oral administration (SCO + POAA3 and SCO + POAA30). Mice in the CON and SCO groups received 0.5% CMC (10 mL/kg, p.o.), while mice in the SCO + DON group received donepezil (3 mg/kg, i.p.) [21, 22]. AA in SLN formulation was then given to mice in the SCO + INAA group intranasally, fifteen *μ*L per nostril (equivalent to a dose of 2.3 mg/kg). The intranasal administration was given to fully awaken mice. The mice in the SCO + POAA3 and SCO + POAA30 groups were orally given AA (3 and 30 mg/kg, respectively). Then, SCO, SCO + DON, SCO + INAA, SCO + POAA3, and SCO + POAA30 mice were injected with scopolamine (3 mg/kg, i.p) [22, 23], while controls were injected with normal saline solution (NSS) (10 mL/kg, i.p). All treatments were given 30 min before the experiments for 10 consecutive days (days 1–10). All mice were performed MWM on days 0–5 and locomotor activity on day 6. On day 10, mice were euthanized by CO_2_. Mice' brains were then quickly removed and dissected for the hippocampus. The brains were snap-freezed in liquid nitrogen and kept in a −80°C freezer for later analysis. The hippocampus was determined by AChE activity and CAT and SOD protein expression. The rest of the brain except the cerebellum was kept for thiobarbituric acid reactive substance (TBARS) assay.

### 2.4. Morris Water Maze

MWM apparatus consisted of a circular water pool (130 cm in diameter and 50 cm in depth) with four visual cues and a platform (8 cm in diameter and 18 cm in height) placed in the center of the northeast quadrant. The water pool was filled with water at a temperature of 22 ± 2°C. On day 0, the platform was visible at 1 cm above the water level, while on days 1–4, the platform was hidden at 1 cm below the water level. On days 0–4, mice were allowed to find the platform for 60 s. Three consecutive trials were performed each day, while mice were started from three quadrants. The escape latency was recorded using a video tracking system (VideoMot2, TSE Systems, Germany), and the average escape latency from the three trials was presented. Mice that failed to reach the platform within 60 s were placed on the platform and allowed to stay for 20 s. On day 5, the probe trial was performed by removing the platform from the pool. A mouse was allowed to find the platform location for 60 s. The time spent in the platform quadrant was recorded.

### 2.5. Open-Field Test

Locomotor activity was performed in a square box (50 × 50 × 40 cm). Mice were placed in the open-field chamber for 30 min following the treatment. The locomotion time was detected using the VideoMot2 system (VideoMot2, TSE Systems, Germany) for 5 min.

### 2.6. Acetylcholinesterase (AChE) Activity

Brain acetylcholinesterase activity was measured using a colorimetric AChE assay kit (Abcam, Cambridge, MA, USA). Brain tissues were homogenized in a lysis buffer on ice and centrifuged at 4°C. The supernatants were then incubated with the acetylthiocholine reaction mixture containing acetylthiocholine and dithiobisnitrobenzoate (DTNB). The reaction product, that is, thiocholine, was then measured using a microplate reader (CLARIOstar®, BMG LABTECH, Germany) at 410 nm after 20 min of incubation.

### 2.7. Western Blotting Analysis

Catalase and SOD protein expression levels were measured using western blot analysis. Brain tissues were lysed in a lysis buffer, and a BCA assay kit (Thermo Fisher Scientific, Rockford, IL, USA) was used to determine the total protein level. In total, 10% SDS-PAGE gel was used to separate protein samples by electrophoresis followed by a PVDF membrane transfer process. Unspecific proteins were blocked with around 5% skim milk. Thereafter, the membrane was incubated with rabbit monoclonal anti-SOD (1 : 5000), anticatalase (1 : 300) (Santa Cruz Biotechnology, CA, USA), and mouse monoclonal anti-GAPDH (1 : 1000) (Millipore, Billerica, MA, USA) antibodies at 4°C overnight. After that, the membrane was incubated with horseradish peroxide (HRP)-conjugated goat anti-rabbit IgG antibodies (1 : 1000) (Millipore, Billerica, MA, USA) or anti-mouse IgG antibodies (1 : 1000) (Santa Cruz Biotechnology, CA, USA) at room temperature for 2 h. The membrane was prepared with a chemiluminescence solution and analysed with a detector of a luminescent image (ImageQuant LAS 4000, GE Healthcare Biosciences, Japan).

### 2.8. Lipid Peroxidation

TBARS assay was used in detecting the lipid peroxidation by measuring malondialdehyde (MDA) levels. The brains were weighed, and a 1 : 5 w/v solution of ice-cold phosphate-buffered saline (PBS) was added at 4°C. Brain tissues were then homogenized and centrifuged at 2000 g for 15 minutes. 100 *μ*L of the samples was incubated in a water immersion at 95 °C for 60 minutes after the addition of approximately 1.5 mL of 20% acetic acid, 1.5 mL of 0.8% TBA, and 0.2 mL of 8.1% SDS. The samples were cooled down for 10 minutes after heating. Then, 2 mL of butanol/pyridine (15 : 1 v/v) was added, and the mixture was centrifuged at 2000 g for 15 minutes. Using a microplate reader (CLARIOstar®, BMG LABTECH, Germany), the supernatants were extracted and measured at 532 nm. 1,1,3,3-tetraethoxypropane (TEP) was utilized to generate a standard curve.

### 2.9. Statistical Analysis

All data were provided as mean ± S.E.M. and analysed using GraphPad Prism version 10.1.0 (GraphPad Software, San Diego, CA, USA). Two-way ANOVA with treatment and time as the main factors was used to analyse the escape latency during the acquisition phase in the Morris water test. For other experiments, the comparison between groups was determined via one-way ANOVA, followed by Fisher's (LSD) post hoc test. The *p* values <0.05 were defined as statistically significant.

## 3. Results and Discussion

### 3.1. Intranasal AA Administration Prevents Scopolamine-Induced Learning and Memory Impairment

**Figure 1:**
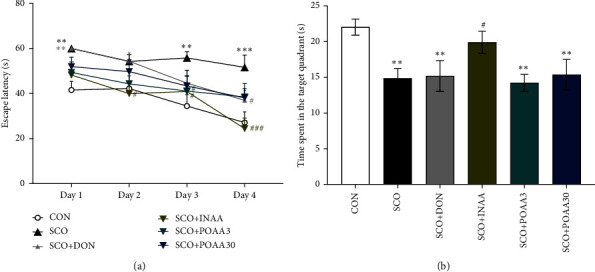
(a) Effects of INAA, POAA3, and POAA30 on the escape latency during the acquisition phase (days 1–4) in the MWM test. Data were provided as mean ± S.E.M. (*n* = 7). ^∗∗^*p* < 0.01, ^∗∗∗^*p* < 0.001 compared with the CON group, ^#^*p* < 0.05, ^###^*p* < 0.001 compared with the SCO group on the same day. (b) Effects of INAA, POAA3, and POAA30 on the time spent in the target quadrant during the probe trial in the MWM test. Data were provided as mean ± S.E.M. (*n* = 7). ^∗∗^*p* < 0.01 compared with the CON group. ^#^*p* < 0.05 compared with the SCO group.

MWM was conducted to evaluate the learning and memory behavior in mice. On day 0, mice received no treatment and performed MWM with a visible platform. It was noted that the mean escape latency at baseline was not different between the groups (*p* > 0.05, one-way ANOVA). On days 1–4, mice received treatment 30 min before the MWM test with a hidden platform. Scopolamine-treated mice had significantly higher escape latency than that of control on days 1, 3, and 4 (*p* < 0.01, *p* < 0.01, and *p* < 0.001, respectively). Donepezil significantly reduced escape latency on day 4 compared to scopolamine treatment alone (*p* < 0.05). Intranasal administration of AA significantly decreased escape latency on days 2, 3, and 4 compared to scopolamine treatment alone (*p* < 0.05, *p* < 0.05, and *p* < 0.001, respectively). POAA3 treatments significantly reduced escape latency on day 3 compared to scopolamine treatment alone (*p* < 0.05). The escape latency of POAA30 mice was not different from that of the control group and the scopolamine group (Figure 1(a)).

The probe trial on day 5 showed that mice in the INAA group spent significantly higher time in the target quadrant than scopolamine-treated mice (*p* < 0.05) (Figure 1(b)). Alternatively, POAA3, POAA30, and donepezil treatment failed to increase the time spent in the target quadrant in the probe trial.

**Figure 2:**
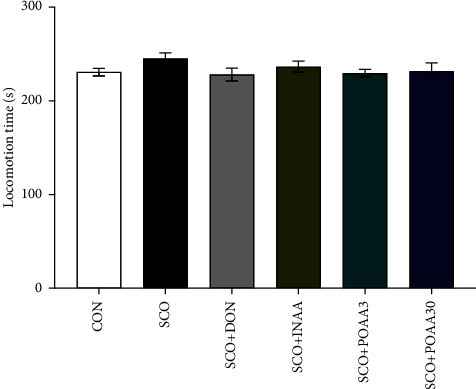
Effect of INAA, POAA3, and POAA30 on locomotor activity in the open-field test. Data were presented as mean ± S.E.M. (*n* = 7).

Locomotor activity was conducted on day 6 to determine the effect of treatment on mice' motor performance. As per the results, no difference was observed in terms of locomotion time among groups (Figure 2), indicating that scopolamine, donepezil, INAA, and POAA do not affect locomotor activity.

AA in solid lipid nanoparticle formulation has been developed for intranasal administration to enhance brain penetration. Our previous study demonstrated successful delivery of AA to the olfactory bulb and hippocampus after a single intranasal administration within 30 minutes [20]. The present study showed that the intranasal administration of AA in SLN prevented scopolamine-induced learning and memory impairment. Acetylcholine plays a vital role in the learning and memory processes in the hippocampus [24]. Learning and memory deficits in patients with AD can be alleviated by acetylcholinesterase inhibitors [6]. In this study, scopolamine, a muscarinic receptor antagonist, induced memory impairments in the MWM test. Intranasal and oral AA treatment as well as donepezil, an AChE inhibitor, can reverse the memory impairment effect of scopolamine. Interestingly, AA given intranasally was noted to produce faster effects than donepezil and oral administration of AA. This result could be due to that intranasal route accelerated drug delivery of AA to achieve the target site of action in the brain. In line with our previous study, nasal administration of AA improved cognitive impairment induced by A*β*_1−42_ in ICR mice [20].

It is noticeable that a low dose of oral AA (3 mg/kg) prevented memory impairment induced by scopolamine, while a high dose of oral AA (30 mg/kg) had no effect. A previous study showed that AA dose-dependently increased the expression of the P-glycoprotein (P-gp) efflux transporter in a concentration-dependent manner [25]. It is likely that low-dose AA did not induce P-gp, thus, AA could reach the effective concentration in the brain and produced an acute effect.

### 3.2. Intranasal AA Administration Inhibits Acetylcholinesterase (AChE) Activity in the Hippocampus

**Figure 3:**
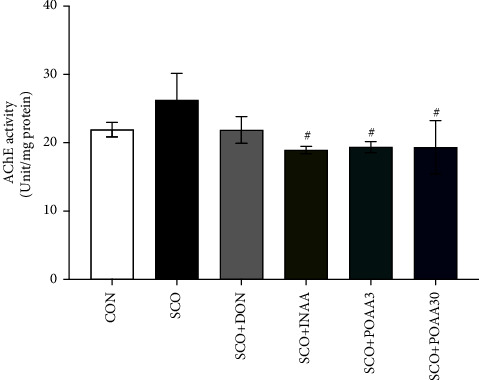
Effect of INAA, POAA3, and POAA30 on acetylcholinesterase (AChE) activity in the hippocampus. Data were provided as mean ± SEM (*n* = 4–6). ^#^*p* < 0.05 compared with the SCO group.

On day 10, mice were terminated 30 min after treatment. The brain AChE activity was then determined to elucidate the mechanism of action of AA. INAA, POAA3, and POAA30 had significantly reduced AChE activity compared to scopolamine treatment alone in the hippocampus (*p* < 0.05) (Figure 3).

Our study demonstrated that AA prevented memory impairments by AChE inhibition. A previous study showed that AChE activity was inhibited by AA using the thin-layer chromatography (TLC) bioautographic method [26]. In addition, raw extract of *Centella asiatica* inhibited AChE activity in SH-SY5Y and AW 264.7 cells, and in the animal model [27]. Previously, the IC_50_ value for AA was determined to be 15.05 *μ*g/mL by *in vitro* analysis, while the binding energy value was found to be −10.27 Kcal·mol^−1^ by *in silico* analysis [13]. Therefore, AA can increase acetylcholine levels through interactions with the active sites of AChE and consequently prevent memory deficits induced by scopolamine. Several studies showed that scopolamine induces memory decline by increasing AChE activity in the mouse brain [28, 29]. Scopolamine inhibited cholinergic transmission by blocking the interaction of acetylcholine (ACh) with muscarinic receptors, leading to an increase in ACh levels in the synaptic cleft [28]. The upregulation of AChE activities might be due to a feedback mechanism to degrade the excess ACh neurotransmitter.

### 3.3. Intranasal AA Administration Elevates Hippocampal Catalase (CAT) Protein Expression and Decreases Brain Lipid Peroxidation

**Figure 4:**
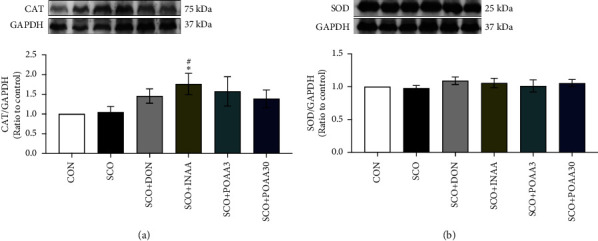
(a) Effect of INAA, POAA3, and POAA30 on catalase (CAT) protein expression in the hippocampus. All data were provided as mean ± S.E.M. (*n* = 7). ^∗^*p* < 0.05 compared with the CON group. ^#^*p* < 0.05 compared with the SCO group. Full blots are presented in the Supplementary data 1. (b) Effect of INAA, POAA3, and POAA30 on superoxide dismutase (SOD) protein expression in the hippocampus. All data were provided as mean ± S.E.M. (*n* = 7). Full blots are presented in the Supplementary data 1.

We investigated the effects of repeated AA administration on the expression of antioxidant enzymes in the hippocampus (Figure 4). It was shown that nasal delivery of AA significantly increased CAT expression levels compared to those of control and scopolamine treatment alone (*p* < 0.05 and *p* < 0.05, respectively) (Figure 4(a)). However, SOD expression was unaltered (Figure 4(b)).

**Figure 5:**
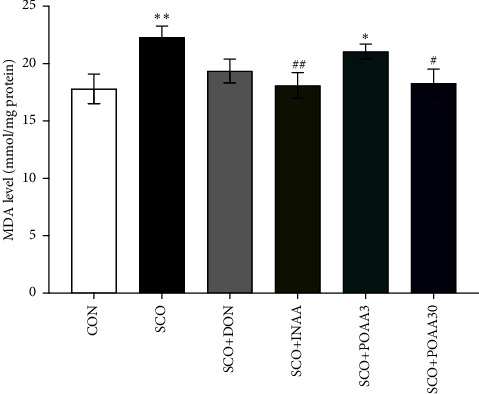
Effect of INAA, POAA3, and POAA30 on MDA levels in mice brains. Data were provided as mean ± S.E.M. (*n* = 6). ^∗^*p* < 0.05, ^∗∗^*p* < 0.01 compared with the CON group. ^#^*p* < 0.05, ^##^*p* < 0.01 compared with the SCO group.

Malondialdehyde (MDA), which is a lipid peroxidation product, was used as a parameter of oxidative stress in brain tissue. Scopolamine and POAA3 treatment significantly increased MDA levels compared to control (*p* < 0.01 and *p* < 0.05, respectively), while INAA and POAA30 administration significantly decreased MDA levels compared to scopolamine treatment alone (*p* < 0.01 and *p* < 0.05, respectively) (Figure 5).

Oxidative stress is one of the main factors involved in the pathological processes in AD [30]. Antioxidants are one of the therapeutic approaches to prevent neurodegeneration in AD [31]. Catalase and SOD are the two main factors of first-line defence antioxidant enzymes in the living tissue [32]. Previous studies indicated that scopolamine-induced oxidative stress in the brains of rodents by decreasing SOD and catalase expression, leading to a decline in MDA levels [33, 34]. A study showed that the antioxidant enzymes were controlled by some transcription factors such as nuclear factor (erythroid-derived 2)-like 2 (Nrf2) and peroxisome proliferator-activated receptor-gamma (PPAR-*γ*) [35]. Scopolamine injection reduced the expression of Nrf2 in the mouse brain [36]. A previous study showed that AA reduced lipid peroxidation by increasing Nrf2 protein expression in chemotherapy-induced neurotoxicity in rats [37] Moreover, AA increased PPAR-*γ* activation in human keloid fibroblasts exposed to TGF-*β*1 [38]. Previous research has demonstrated the antioxidant properties of AA in a rodent model of memory loss produced by quinolinic acid and aluminium chloride (AlCl_3_) by increasing the expression of superoxide dismutase (SOD), catalase, and glutathione and decreasing lipid peroxidation [10, 39]. In line with our study, repeated intranasal delivery of AA increased the expression of catalase in the hippocampus and subsequently reduced lipid peroxidation, suggesting the benefit of AA to increase antioxidants in the normal brain.

## 4. Conclusions

**Figure 6:**
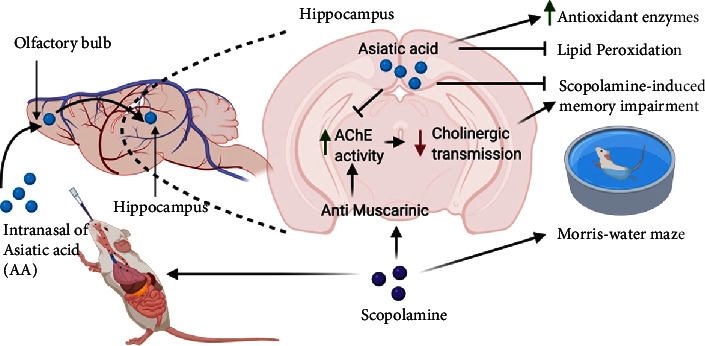
Schematic diagram of the proposed mechanisms of AA by nasal delivery in mice.

In summary, our study showed that intranasal AA administration protected against scopolamine-induced learning and memory impairment in mice. The effect of intranasal administration of AA was faster than AA oral administration. Repeated intranasal administration of AA inhibited acetylcholinesterase activity, elevated antioxidant enzyme expression, and decreased lipid peroxidation in the brain (Figure 6). This study suggests that AA can be a potential treatment for AD.

## Data Availability

Data will be made available upon request from the corresponding author.

## Supplementary Materials

Supplementary Materials Supplementary data 1: original full-length of catalase (CAT) and superoxide dismutase (SOD) blot for Figures 4(a) and 4(b).
