# Supplementary material for: Nasal Delivery of Asiatic Acid Ameliorates Scopolamine-Induced Memory Dysfunction in Mice
Source: Adv Pharmacol Pharm Sci. 2024 Sep 9;2024:9941034. doi: 10.1155/2024/9941034 (PMC11405110; doi:10.1155/2024/9941034)

Sam 1 SOD CAT (2nd running half chemi) after washing

75kDa	7414.995	9734.652	8022.066	13536.72	11219.82	10084.89	6389.924
64kDa	5992.057	6476.53	6074.329	7584.874	7959.874	8978.986	8990.128

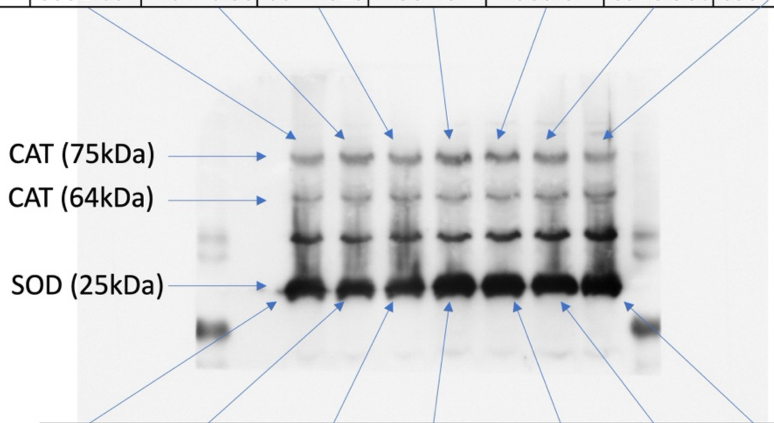

14890.46	13833.66	12079.22	16724.1	16142.39	15272.13	14670.46

Sam 1 GAPDH 2nd running half chemi

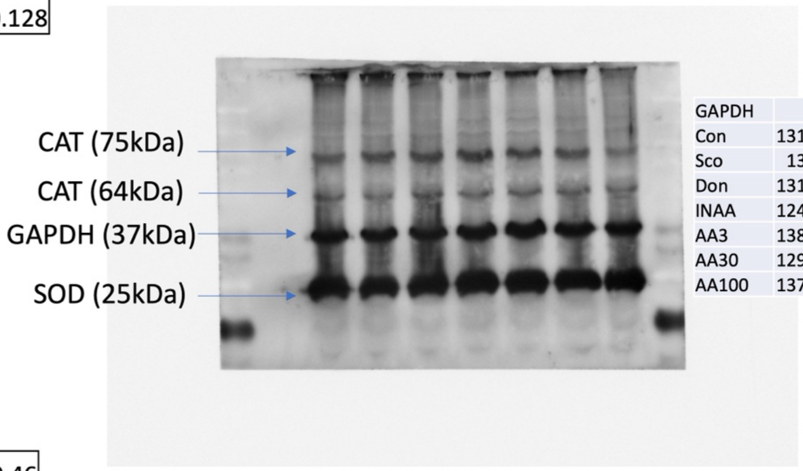

GAPDH	
Con	13170.79
Sco	13823.6
Don	13135.95
INAA	12445.68
AA3	13842.16
AA30	12948.85
AA100	13740.97

HPC

Sam 2 SOD CAT (2nd running half chemi) after washing

75kDa	7275.095	10088.77	7683.388	13777.38	11478.65	10380.89	6269.267
64kDa	6215.22	5366.886	6194.936	6351.258	6721.572	7053.2	8113.442

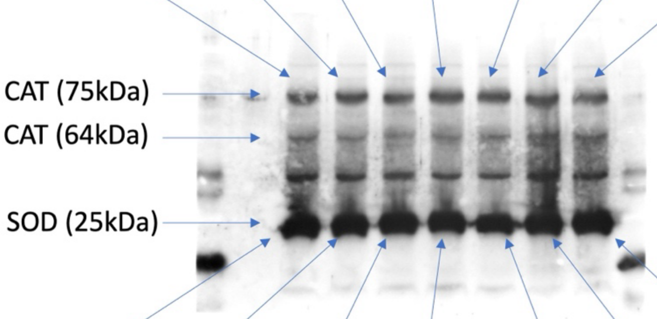

14749.78	16368.8	16176.97	16734.92	17860.36	15122.56	18553.38

Sam 2 GAPDH 2nd running

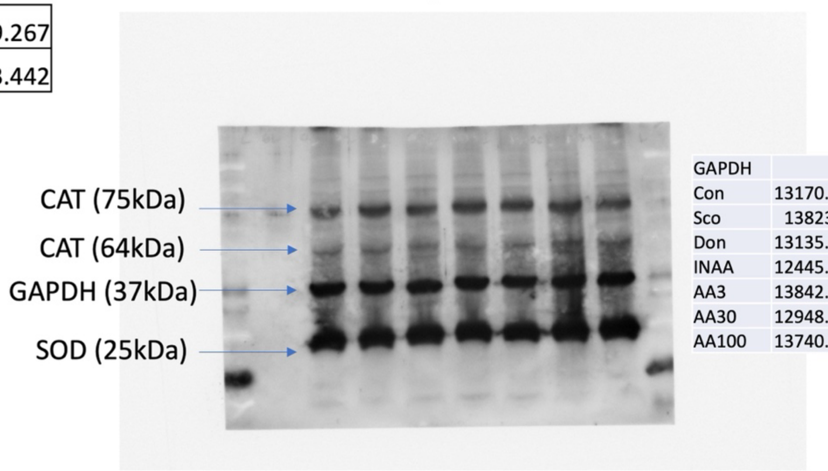

GAPDH	
Con	13170.79
Sco	13823.6
Don	13135.95
INAA	12445.68
AA3	13842.16
AA30	12948.85
AA100	13740.97

HPC

Sam 3 SOD CAT (1st running half chemi)

75kDa	8410.865	8515.693	13246.76	11408.01	7940.723	7140.087	7714.735
64kDa	3039.167	3431.61	3916.853	4190.803	4840.137	5337.38	6451.744

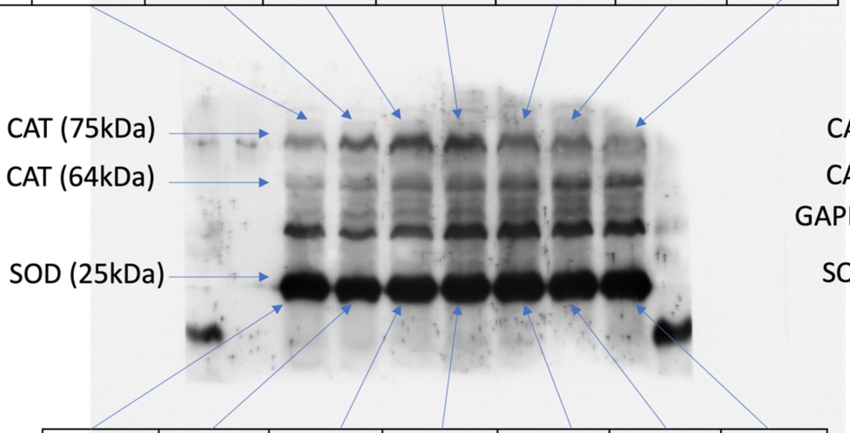

30222.1	26656.96	26943.23	23888.39	24961.93	26803.05	27905.27

Sam 3 GAPDH (2nd running half chemi)

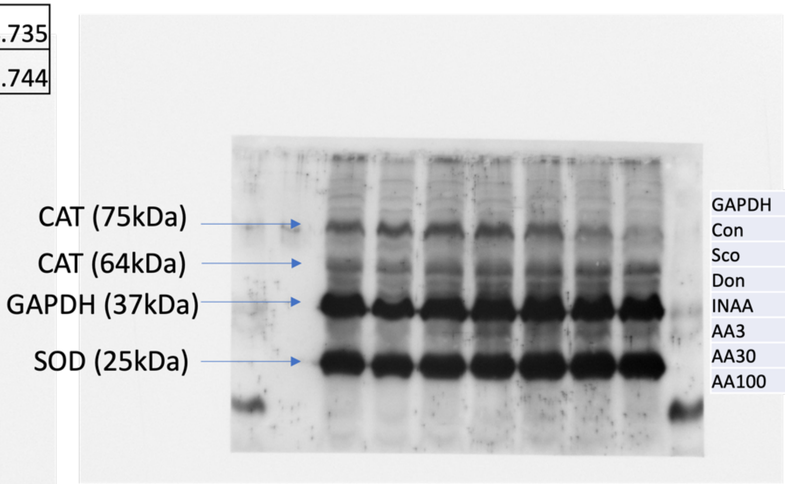

GAPDH	
Con	19122.79
Sco	19143.16
Don	17315.38
INAA	14259.89
AA3	14082.25
AA30	15391.92
AA100	17971.74

HPC

Sam 4 SOD CAT (2nd running half chemi)

75kDa	6971.53	7028.288	8669.238	12598.23	13028.82	10797.23	9176.016
64kDa	5207.581	6146.581	5963.924	6982.288	7535.886	6582.815	6583.563

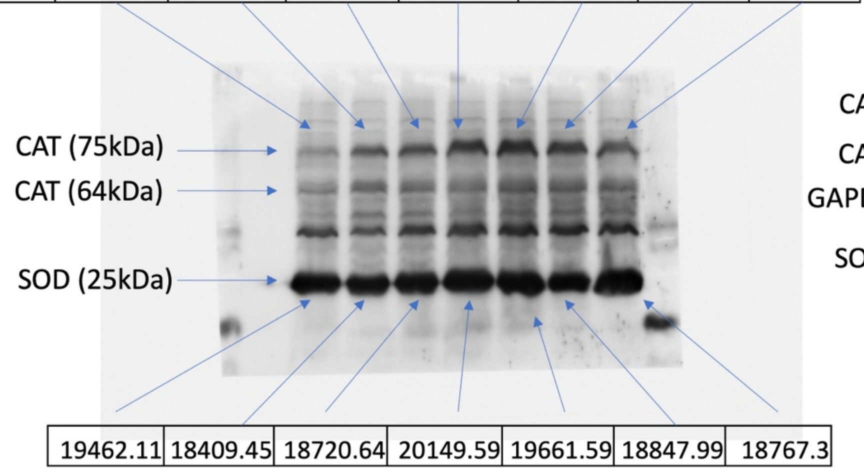

19462.11	18409.45	18720.64	20149.59	19661.59	18847.99	18767.3

Sam 4 GAPDH (1st running half chemi)

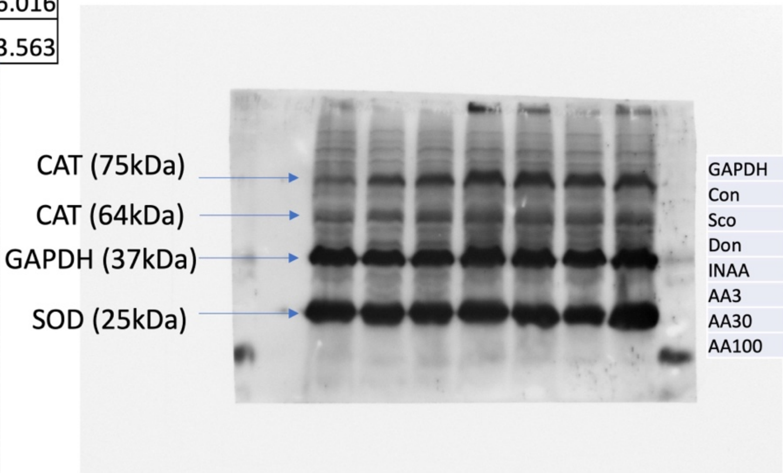

GAPDH	
Con	18373.16
Sco	17598.89
Don	14637.48
INAA	15222.49
AA3	14826.71
AA30	14622.62
AA100	15263.69

HPC

HPC SOD CAT GAPDH sam 5

75 kDa	3831.953	3538.146	8080.744	9007.309	10340.41	6582.752	5384.083
64 kDa	7783.137	8070.673	8206.844	8004.288	7535.045	5985.945	7635.045

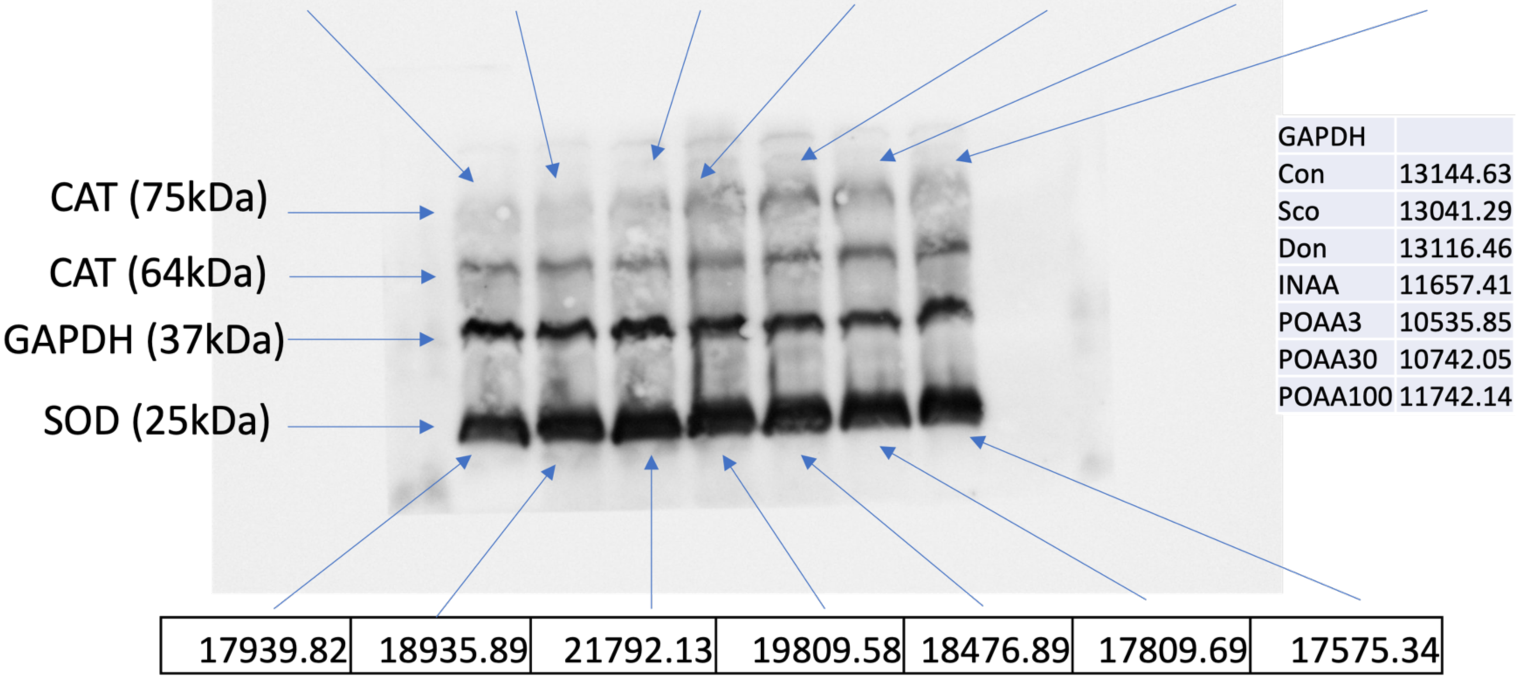

GAPDH	
Con	13144.63
Sco	13041.29
Don	13116.46
INAA	11657.41
POAA3	10535.85
POAA30	10742.05
POAA100	11742.14

17939.82	18935.89	21792.13	19809.58	18476.89	17809.69	17575.34

*When running this sample, SOD, CAT and GAPDH were probe together. Therefore, it has only one day data.

HPC sam 6 SOD CAT (1st running half chemi 1min)

75kDa	3498.418	3459.125	2505.276	3604.489	3857.752	4307.803	3438.439
64kDa	13315.87	4329.125	9615.317	5894.731	5163.832	5428.66	5380.296

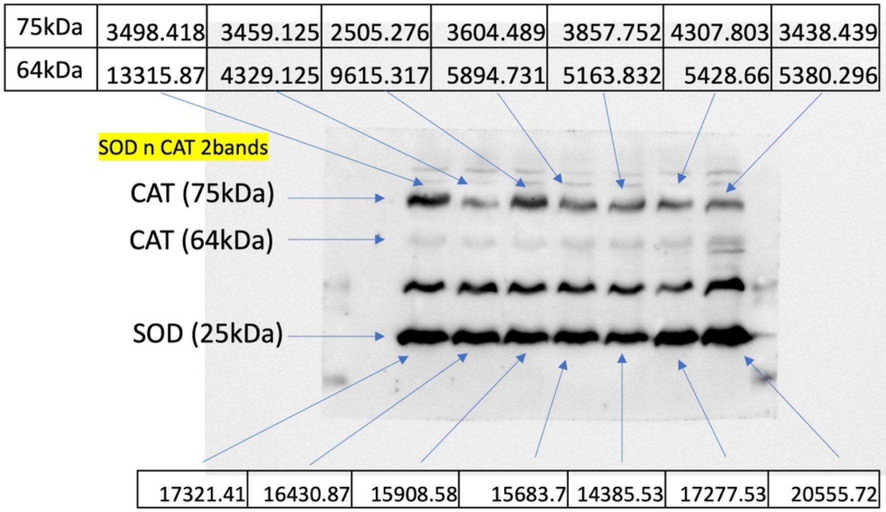

17321.41	16430.87	15908.58	15683.7	14385.53	17277.53	20555.72

HPC sam 6 GAPDH (1st running half chemi)

12611.56	12611.56	12611.56	12611.56	12611.56	12611.56	12611.56

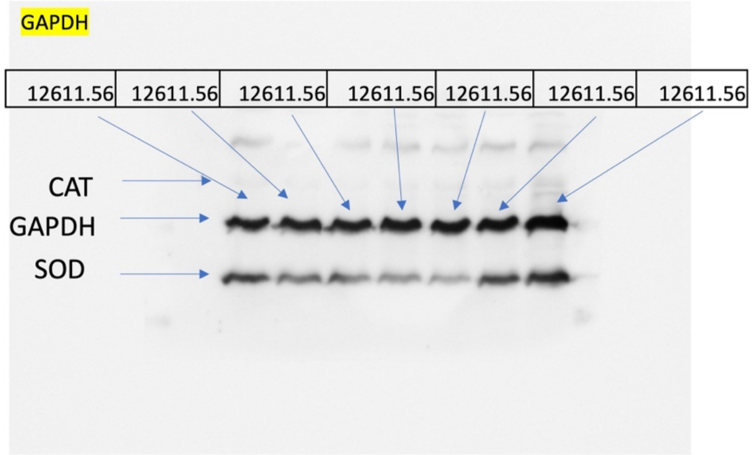

HPC sam 7 SOD CAT (1st running half chemi 1min)

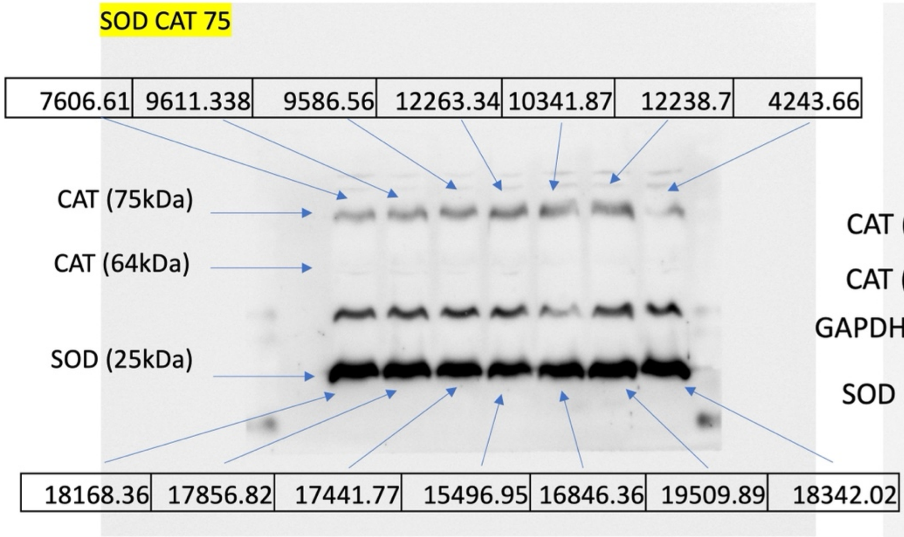

HPC sam 7 GAPDH (1st running half chemi)

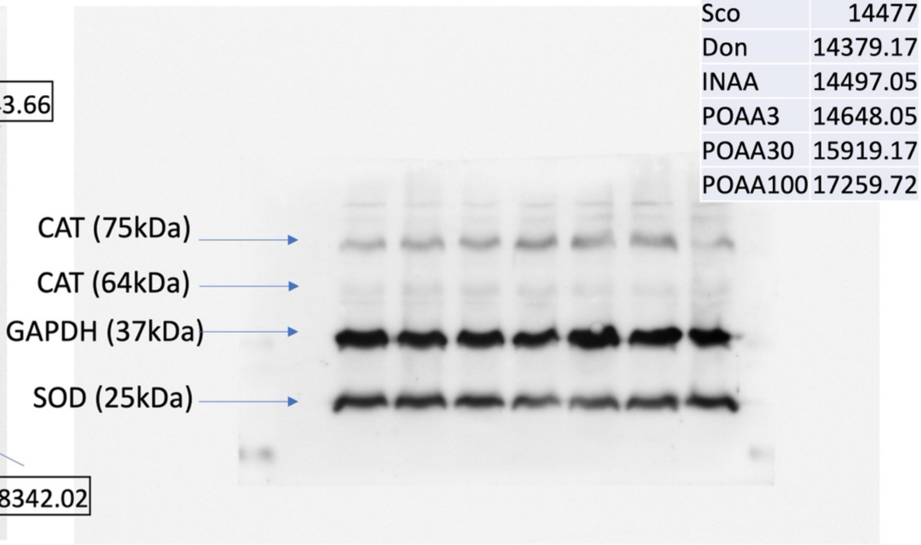

GAPDH	
Con	12611.56
Sco	14477
Don	14379.17
INAA	14497.05
POAA3	14648.05
POAA30	15919.17
POAA100	17259.72

* CAT 64 kDa band cannot measure. Therefore, HPC sam 7 SOD CAT (2nd running half chemi 5min) from next slide is used to measure CAT 64 kDa.

HPC sam 7 SOD CAT (2nd running half chemi 5min)

CAT 64

4818.823	4790.773	5063.53	4958.409	6113.045	6282.137	4526.43

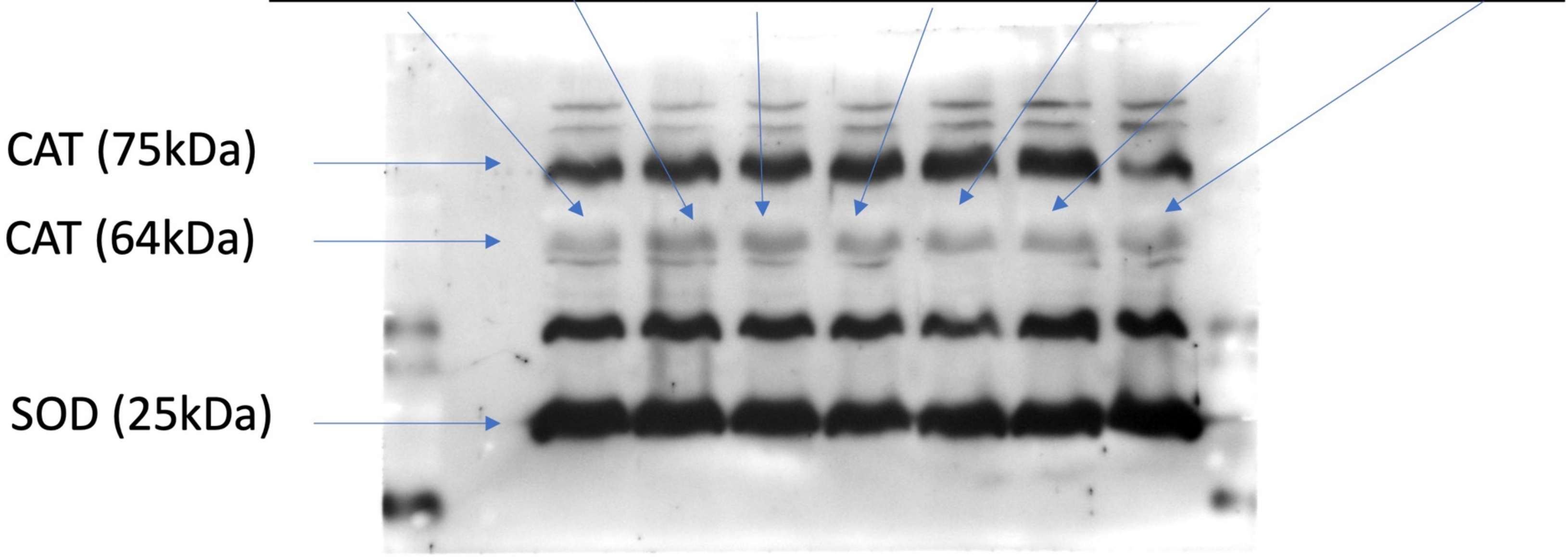

Supplement: Supplementary Materials — Supplementary data 1: original full-length of catalase (CAT) and superoxide dismutase (SOD) blot for Figures 4(a) and 4(b). [file 9941034.f1.pdf]
